# Retinol, Carotenoid, and Tocopherol Intake and Status, and the Risk of Islet Autoimmunity and Type 1 Diabetes: The Environmental Determinants of Diabetes in the Young Study

**DOI:** 10.1002/dmrr.70196

**Published:** 2026-06-26

**Authors:** Leena Hakola, Joanna L. Clasen, Ulla Uusitalo, Carin Andrén Aronsson, Sandra Hummel, Markus Mattila, Sari Niinistö, Jorma Toppari, Anette‐G. Ziegler, William A. Hagopian, Beena Akolkar, Marian J. Rewers, Richard A. McIndoe, Åke Lernmark, Jeffrey P. Krischer, Jill M. Norris, Iris Erlund, Suvi M. Virtanen, the TEDDY Study Group

**Affiliations:** ^1^ Unit of Health Sciences Faculty of Social Sciences Tampere University Tampere Finland; ^2^ Tampere University Hospital Wellbeing Services County of Pirkanmaa Tampere Finland; ^3^ Department of Public Health Finnish Institute for Health and Welfare Helsinki Finland; ^4^ Health Informatics Institute Morsani College of Medicine University of South Florida Tampa Florida USA; ^5^ Department of Clinical Sciences Lund University Malmö Sweden; ^6^ Department of Pediatrics Skåne University Hospital Malmö Sweden; ^7^ Institute of Diabetes Research Helmholtz Zentrum München German Research Center for Environmental Health Munich‐Neuherberg Germany and Forschergruppe Diabetes at Klinikum rechts der Isar School of Medicine and Health Technical University of Munich, Munich, and Forschergruppe Diabetes e.V. Neuherberg Germany; ^8^ Department of Pediatrics Turku University Hospital Turku Finland; ^9^ Institute of Biomedicine Research Centre for Integrative Physiology and Pharmacology University of Turku Turku Finland; ^10^ Pacific Northwest Research Institute Seattle Washington USA; ^11^ National Institute of Diabetes & Digestive & Kidney Diseases Bethesda Maryland USA; ^12^ Barbara Davis Center for Childhood Diabetes University of Colorado Aurora Colorado USA; ^13^ Center for Biotechnology and Genomic Medicine Medical College of Georgia Augusta University Augusta Georgia USA; ^14^ Department of Epidemiology Colorado School of Public Health University of Colorado Aurora Colorado USA; ^15^ Institute for Nutrition and Health Research Helsinki Finland; ^16^ Tampere Center for Child Adolescent and Maternal Health Research (TamCAM) Tampere University and Tampere University Hospital Tampere Finland; ^17^ Department of Pediatrics Tampere University Hospital Tampere Finland

**Keywords:** birth cohort, carotenoids, diabetes mellitus, type 1, retinol, tocopherols, vitamin A, vitamin E

## Abstract

**Aims:**

To study the associations of dietary intake of A and E vitamins, as well as plasma retinols, carotenoids, and tocopherols in relation to development of islet autoimmunity and progression to T1D.

**Materials and Methods:**

The Environmental Determinants of Diabetes in the Young (TEDDY) Study followed 7659 newborns with genetic susceptibility to T1D for 6 years in the USA, Finland, Germany, and Sweden. Dietary vitamin intake was assessed repeatedly with 3‐day food‐records in full cohort at ages 6 months to 6 years. Plasma retinols, carotenoids, and tocopherols were analysed in a nested case‐control setting with 359 children with islet autoimmunity and 1033 matched controls.

**Results:**

In the full cohort analyses, dietary intake of retinol, *β*‐carotene, and vitamin E was not associated with the risk of islet autoimmunity or progression to T1D. Further, none of the plasma retinol, carotenoid, and tocopherol biomarkers were associated with islet autoimmunity or T1D in the full nested case‐control analyses. We observed effect modification by country, breastfeeding, sex, and follow‐up time for both intake and biomarkers of vitamins on the risk of islet autoimmunity or T1D, and some subgroup associations. Finally, a plasma carotenoid metabolite (likely zeinoxanthin) (OR 0.61, 95% CI 0.39, 0.95, *p* = 0.03) and *γ*‐carotene at 6 months (OR 0.65, 95% CI 0.45, 0.94, *p* = 0.02) were inversely associated with the odds of developing GADA‐first.

**Conclusions:**

Retinol, carotenoids and tocopherols were not consistently associated with islet autoimmunity. This study adds to the understanding of factors and their interactions related to T1D development.

**Abbreviations**
CI: confidence interval
CrI: credible interval
DIPP: Finnish Type 1 Diabetes Prediction and Prevention
FDR: first‐degree relative with type 1 diabetes
GADA: glutamic acid decarboxylase autoantibodies
IAA: insulin autoantibodies
IA‐2A: insulinoma antigen‐2 autoantibodies
T1D: type 1 diabetes
TEDDY: the environmental determinants of diabetes in the young

## 1 Introduction

Type 1 diabetes (T1D) is alleged to develop due to a combination of genetic predisposition and environmental or immunological triggers that lead to an autoimmune process characterised by circulating islet autoantibodies [1]. The Environmental Determinants of Diabetes in the Young (TEDDY) Study, a prospective birth cohort of children at increased genetic risk, aims to identify environmental causes of T1D [2]. In addition to environmental triggers that may initiate or facilitate the disease process, there might be environmental factors that attenuate or reverse this process. Potential candidates include vitamins with antioxidant properties or roles in immune function. In TEDDY, we previously observed that intake of some dietary B vitamins [3] as well as plasma levels of 25‐hydroxyvitamin D [25(OH)D] [4] and ascorbic acid [5] were inversely associated with at least one type of islet autoimmunity.

Few studies have investigated the role of vitamins A and E or related metabolites in the risk of islet autoimmunity and T1D. A recent finding Finnish Type 1 Diabetes Prediction and Prevention (DIPP) suggest that higher vitamin E intake in childhood is associated with decreased risk of islet autoimmunity and T1D [6]. However, A and E‐vitamin related biomarkers serum α‐ and β‐carotene [7] and α‐ and γ‐tocopherol [8] showed no such association. However, another prospective study in children suggested a potential inverse association between serum *α*‐tocopherol and risk of T1D [9]. To our knowledge, associations of plasma/serum retinol or carotenoids other than *α*‐ and *β*‐carotene with the risk of islet autoimmunity and T1D have not been studied before.

In TEDDY, we previously reported a direct association between dietary intake of *β*‐carotene and plasma *β*‐carotene, as well as between dietary vitamin E and plasma *α*‐tocopherol and *γ*‐tocopherol [10]. For retinol, dietary intake did not associate with plasma level [10]. The intake of any breastmilk versus no breastmilk was associated with lower retinol and *γ*‐tocopherol concentrations [10]. As many factors other than dietary intake (e.g., absorption and homoeostasis) affect the biomarker levels, the intake and status can be considered as partly different exposures with partly different mechanisms of action in the body.

Our aim was to study the dietary intake of A and E vitamins, as well as plasma retinols, carotenoids, and tocopherols in relation to the development of islet autoimmunity and progression to T1D in the prospective multinational TEDDY cohort.

## 2 Materials and Methods

### 2.1 Study Population and Study Designs

TEDDY is a prospective observational birth cohort study designed to identify environmental triggers of T1D [11]. The enrolment of 8676 newborn children was carried out in clinical centres located in the USA (Washington, Colorado, and Georgia), Finland, Sweden, and Germany, from September 2004 through February 2010. The study included children with genetic and familial susceptibility to T1D. Eligibility criteria for initial contact was one of the following HLA‐DR genotypes: HLA‐DR3/4; HLA‐DR4/4; HLA‐DR4/8, and HLA‐DR3/3. Infants with HLA‐DR genotypes HLA‐DR4/1, HLA‐DR4/13, HLA‐DR4/9, and HLA‐DR3/9 were included only if they had a first‐degree relative with T1D (FDR) [12].

For all participants, written informed consent was obtained from a parent or primary caretaker for genetic screening, and separate consent was obtained for participation in the prospective follow‐up. The study was approved by the local Institutional Review or Ethics Boards at each site and is monitored by an External Evaluation Committee formed by the National Institutes of Health.

Follow‐up visits at the study clinics were scheduled every 3 months between ages 3–48 months and biannually thereafter. However, children with islet autoantibodies were followed up every 3 months regardless of the age.

In the present study, we utilise two study settings (Supporting Information S1; Figure S1): (1) a cohort study to examine the associations of dietary intake with risk of islet autoimmunity and progression to T1D, and (2) two nested case‐control studies to study dietary biomarkers and the risk of (2a) islet autoimmunity (359 case children); and (2b) T1D (104 case children) [13]. Most case children with T1D were also case children with islet autoimmunity (Supporting Information S1; Figure S1). Up to three matched controls were selected for each case child. Children were matched for FDR, clinical centre, and sex. The nested case–control study selection was based on the islet autoantibody and T1D data collected as of 31 May 2012 [13]. As the nested case‐control studies covered the first 6 years of life, for comparability, the follow‐up in the cohort was restricted to 6 years of age as well.

### 2.2 Outcomes

Islet autoimmunity status was based on blood samples that were obtained at each study visit to screen for autoantibodies against insulin (IAA), glutamic acid decarboxylase (GADA), and insulinoma antigen‐2 (IA‐2A) [11].

In the cohort and in the nested case‐control design, islet autoimmunity was defined as the presence of at least one autoantibody (IAA, GADA, or IA‐2A) in two or more consecutive samples confirmed in two laboratories. We also studied outcomes IAA‐first, and GADA‐first among those who had any IA, as well as multiple islet autoimmunity defined as repeated positivity to at least two autoantibodies. T1D was defined using the American Diabetes Association criteria [14] both in the cohort and nested case‐control study. In the cohort, the risk of T1D was studied among children with persistent islet autoantibody positivity who had first positivity between ages 6 months and 6 years (progression from islet autoimmunity to T1D).

### 2.3 Exposures

Total vitamin intakes were calculated from all foods, drinks (including breastmilk), and dietary supplements reported in the 3‐day food records collected at the ages of 6, 9, and 12 months, and biannually until the age of 6 years. Primary caretakers were trained during the 3‐month clinic visit to keep 3‐day food records of the child's dietary intake. Participants were instructed to fill out the 3‐day food record within 10 days prior to the next clinic visit with two weekdays and one weekend day. At each clinic visit, the food records were reviewed by trained study personnel together with the primary caretaker. The amount of breastmilk was estimated based on the difference between estimated total energy expenditure [15] and energy intake from other foods and drinks among the breastfed children for visits < 36 months. A description of the dietary assessment methods used in TEDDY has been described in detail before [16].

Food record data entry was done by trained personnel. Calculation of vitamin intake was based on four national food composition databases: FINELI (Finland), LEBTAB (Germany), NFA Food Composition Database (Sweden), and Nutrition Data System for Research software developed by the University of Minnesota Nutrition Coordinating Centre (NCC) (USA).

After the harmonisation efforts of the food composition databases, TEDDY researchers concluded that of the vitamin A, carotenoid, and vitamin E variables available in TEDDY, the comparable forms between all four countries are the following: **retinol** (calculated as retinol equivalents = retinol + 0.167 × *β* − carotene equivalents), **β ‐carotene**, and **vitamin E** (calculated as *α*‐tocopherol equivalents) [17], and these are used as exposure variables in this study.

Plasma samples for A and E vitamin‐related biomarkers were collected in the years 2004 through 2012 at age 6 months and annually from 1 year to 6 years of age, up to the outcome age for cases and the outcome age of the matched case for controls. Children living far away from the nearest TEDDY study centre followed the long‐distance sample collection protocol, in which blood samples were collected by the child's paediatrician and transported to a TEDDY study centre within 24 h. Samples were aliquoted into dedicated, barcoded (Symnol LS 2208), and colour‐coded cryovials as determined by the type of analyses to be performed [18]. Preservatives were added at the initial collection stage to enhance the stability of the analyte. The blood samples were then shipped frozen to the NIDDK Repository and immediately stored at −80°C. Quality control (QC) plasma samples that appeared identical to actual subject samples were incorporated into the overall sample management in a blinded manner to determine intra‐assay variability.

All dietary biomarker measurements were performed at the Department of Government Services (Biomarkers laboratory), Finnish Institute for Health and Welfare. The mean storage time from sample collection to laboratory analysis was 64 months.

Plasma retinols, carotenoids, and tocopherols were analysed by reversed‐phase HPLC [19] after precipitation of proteins with ethanol followed by extraction with hexane. Retinol and carotenoids were determined by HPLC with multiwavelength detection and *α*‐tocopherol and *γ*‐tocopherol were determined by HPLC and fluorescence detection using Agilent equipment (Agilent Technologies Inc., Santa Clara, CA, USA). Analyte identification was based on comparing retention times and absorbance spectra with those of commercial standards, except for minor carotenoid metabolites, named as “C” metabolites. The carotenoid metabolites were tentatively identified by comparing retention times and absorbance spectra with those reported in previous literature: C2 (lutein metabolite), C6 (rubixanthin), and C7 (zeinoxanthin). The Cholesterol Assay was used to analyse total cholesterol enzymatically, using the ARCHITECT ci8200 analyser (Abbott Laboratories, Abbott Park, IL, USA). Intra‐assay precision [coefficient of variation (CV)] of the methods was 4.6%–5.6% for retinol, *β*‐carotene, *α*‐tocopherol, and *γ*‐tocopherol, < 11% for other carotenoids and carotenoid metabolites, and < 0.6% for cholesterol, for control samples used for validation of the method. Inter‐assay precision (CV) of QC samples included in each batch during analysis of case‐control samples were as follows, retinol (7.3% ± 0.8%), *β*‐carotene (6.0% ± 2.4%), *α*‐tocopherol (9.2% ± 3.1%), *γ*‐tocopherol (6.0% ± 0.2%), and cholesterol (1.40% ± 0.03%) [mean ± standard deviation (SD)].

Statistical analysis of QC sample values indicated there was no significant batch effect on any of the analytes. To achieve the best possible accuracy, the values of the calibrators were compared with reference standards. Certified reference materials were purchased from the National Institute of Standards and Technology. For standardising measurements and for ensuring analytical reliability, the laboratory took part in external quality assessment programs. For cholesterol, the laboratory participated in the Lipid Standardisation Programme organised by the CDC (Atlanta, GA) and an external quality assessment scheme organised by Labquality (Helsinki, Finland). For carotenoids and tocopherols, the laboratory participated in the Micronutrients Measurement Quality Assurance Programme arranged by the National Institute of Standards and Technology.

### 2.4 Statistical Methods

#### 2.4.1 Cohort—Islet Autoimmunity

Follow‐up began at age 6 months and ended at age 6 years, islet autoimmunity seroconversion age, or study withdrawal. Nutrient intakes were excluded when identified as outliers according to the IQR method with a scale factor of 5 (1.3% of all food records) [20], were adjusted for energy intake using the density method (units of intake per 1000 kcal of energy), and then scaled to a standard deviation (SD) of 1 within each visit age. Bayesian joint models were used that combined longitudinal intake data and time‐to‐event islet autoimmunity data into a single model, with separate joint models run for each nutrient. Linear mixed effects models were used for the longitudinal submodels, with a diagonal structure for the variance‐covariance matrix, and restricted cubic splines were used to estimate the fixed and random effects of time with knots placed at the fifth, 35th, 65th, and 95th percentiles [21]. Survival submodels were built using the structure of the Cox model with the baseline hazard estimated by B splines with 10 segments. A current value association structure was used, so for a given point in time, a 1 SD increase in intake was assessed in relation to risk of islet autoimmunity at that same time point. The posterior distribution was sampled with a Markov chain Monte Carlo (MCMC) algorithm with 3 chains, and chain convergence was assessed with Rhat values and visual inspection of trace plots. The survival submodels were adjusted for sex, FDR (yes, no), HLA (DR3/4, other), and country. Breastfeeding status at 6 months was added to the model for outcome of any islet autoimmunity. Energy intake was included as a second longitudinal outcome in each joint model. Models were checked for an interaction with age to determine if the association between intake and islet autoimmunity risk changed over time, and these models were compared to models without time interaction using the WAIC values. Competing risk joint models [22] were used for individual first appearing autoantibodies: IAA, GADA, or IA‐2A/multiple. Interactions were assessed between intake and country, sex, HLA, and breastfeeding status. Equivalent joint models were employed to assess the associations between nutrient intake and risk of multiple islet autoimmunity. In a sensitivity analysis, Cox proportional hazards models with intake as a linear variable and with intake modelled using restricted cubic splines were compared to assess potential non‐linearity.

#### 2.4.2 Cohort—Progression

Cox proportional hazards models were used to examine the associations between nutrient intake at the islet autoimmunity seroconversion visit (or up to 12 months prior) and risk of progression from islet autoimmunity to T1D, with follow‐up for T1D extending to age 6 years. The exposure variables were the energy density‐adjusted nutrient intakes. Models with time since islet autoimmunity seroconversion as the underlying timescale were adjusted for sex, FDR, HLA, energy intake, first appearing autoantibody, and islet autoimmunity seroconversion age, and the baseline hazard was stratified by country. The assumption of proportional hazards was checked with plots of Schoenfeld residuals. Interactions were assessed between intake and each covariate.

#### 2.4.3 Nested Case‐Control

Each biomarker was log2‐transformed. Then the average across all visits was calculated and used as a summary measure. Additionally, the value from the 6‐month visit was used as an infancy. Pearson correlations between biomarkers were calculated, separately for each visit age. Conditional logistic regression models were used to compare islet autoimmunity and T1D cases with matched control subjects. Time‐varying potential confounders were adjusted for with linear mixed models of each biomarker regressed on the potential confounder, using the residuals as the exposure in the primary models. All conditional logistic regression models were adjusted for HLA, then additional adjustments were progressively added: cholesterol and breastfeeding status at age 6 months (any breastfeeding, yes/no), then weight‐for‐age z‐score, parental smoking status (either parent smokes, yes/no), antibiotic use (within the same month as the blood draw, yes/no), gastrointestinal infection (within 14 days prior to blood draw, yes/no), respiratory infection (within 14 days prior to blood draw, yes/no), and coeliac disease status (previously diagnosed, diagnosed within the following year, healthy). Covariates were chosen based on a previous analysis of factors associated with dietary biomarker concentrations in TEDDY [10] and the observed correlations between cholesterol and other biomarkers. Sensitivity analyses for islet autoimmunity and T1D were conducted with an additional adjustment for long‐distance protocol [10] and with exclusion of plasma samples that failed in laboratory's internal quality control. Additionally, the associations of islet autoimmunity and T1D with the change in biomarker concentration over time were assessed with Bayesian linear mixed models with random intercepts and slopes, and the individual‐specific slope was used as the exposure in conditional logistic regression models. Log‐linearity was assessed with the supremum test using cumulative Martingale residuals to determine if it was reasonable to treat biomarker concentrations as continuous variables [23]. Interactions between biomarker concentration and country, sex, and breastfeeding status were examined. For the analysis assessing risk of T1D, a sensitivity analysis was run excluding the last 18 months of follow‐up prior to case T1D age to allow for the possibility that subclinical metabolic changes may precede diagnosis.

Data wrangling was done in SAS, version 9.4, and analyses were conducted in *R* version 4.4.3 [24] with the packages JMbayes2 version 2_0.5‐0 [25], survival version 1_3.8‐3 [26], and brms version 2.22.0 [27].

## 3 Results

### 3.1 Cohort Study

Of the 8676 children enroled in the study, 1017 children were excluded due to HLA ineligibility (*n* = 125), indeterminate autoantibody status (*n* = 53), autoantibody follow‐up ended before the age of 6 months (*n* = 496), no food record data (*n* = 284), or no breastfeeding data (59), resulting in 7659 participants in the cohort analyses (Supporting Information S1; Figure S1). The cohort study population is described in Table 1. In the cohort, the median outcome age was 2.3 years for islet autoimmunity (*n* = 574), 1.7 years for IAA‐first (*n* = 244), 2.8 years for GADA‐first (*n* = 222) and 2.5 years for multiple islet autoimmunity (*n* = 340). Among the 508 islet autoimmunity positive children in the progression cohort, 140 (38%) developed T1D at a median age of 3.2 years with a median 1.9‐year follow‐up from first islet autoimmunity positivity.

**TABLE 1:** Characteristics of TEDDY participants in the cohort study by country.

	USA (*n* = 3164)	Finland (*n* = 1674)	Germany (*n* = 486)	Sweden (*n* = 2335)	Total (*n* = 7659)
	*n* (%)	*n* (%)	*n* (%)	*n* (%)	*n* (%)
Sex					
Female	1555 (49)	812 (49)	239 (49)	1151 (49)	3757 (49)
Male	1609 (51)	862 (51)	247 (51)	1184 (51)	3902 (51)
HLA^a^					
DR3/4	1272 (40)	556 (33)	190 (39)	971 (42)	2989 (39)
Other	1892 (60)	1118 (67)	296 (61)	1364 (58)	4670 (61)
FDR^b^					
No	2794 (88)	1493 (89)	301 (62)	2150 (92)	6738 (88)
Yes	370 (12)	181 (11)	185 (38)	185 (8)	921 (12)
Breastfed at 6 months					
No	1420 (45)	527 (31)	196 (40)	884 (38)	3027 (40)
Yes	1744 (55)	1147 (69)	290 (60)	1451 (62)	4632 (60)

Abbreviations: GADA, glutamic acid decarboxylase; IAA, islet autoantibodies against insulin; IA‐2A, insulinoma antigen‐2.

^a^ HLA, human leucocyte antigen.

^b^ FDR, first degree relative with T1D.

#### 3.1.1 Vitamin Intake

The median energy‐adjusted intake of retinol, *β*‐carotene, and vitamin E peaked at an early age and remained stable during later childhood (Supporting Information S1; Figures S2 and S3). Intake profiles were somewhat different depending on breastfeeding status and country (Supporting Information S1; Figure S3).

Dietary intake of retinol, *β*‐carotene, and vitamin E was not associated with any of the islet autoimmunity outcomes adjusted for sex, FDR, HLA, and country (Table 2). There were no associations with vitamin intake and islet autoimmunity after further adjustment for breastfeeding. Time‐interaction analyses suggest that a higher intake of vitamin E may be associated with a decreased risk of islet autoimmunity at an early age, up to the age of 1.5 years but not after that (Supporting Information S1; Figure S4). No such time interaction was seen for retinol or *β*‐carotene intake and islet autoimmunity.

**TABLE 2:** Associations between dietary intake of retinol, *β* ‐carotene, and vitamin E and risk of type 1 diabetes (T1D)‐related outcomes in children aged 6 months to 6 years, the TEDDY cohort.

*n* outcome/cohort	Any IA	IAA‐first	GADA‐first	Multiple IA	Progression to T1D
	574/7659	244/7659	222/7659	340/7659	140/508
	HR (95% CrI)^a^	HR (95% CrI)^a^	HR (95% CrI)^a^	HR (95% CrI)^a^	HR (95% CI)^b^
Retinol	1.19 (0.95, 1.52)	1.09 (0.73, 1.61)	1.23 (0.81, 1.82)	1.17 (0.86, 1.54)	1.02 (0.98, 1.06)
*β*‐carotene	1.06 (0.84, 1.52)	1.05 (0.72, 1.49)	1.16 (0.79, 1.64)	0.94 (0.68, 1.22)	1.004 (0.997, 1.06)
Vitamin E	0.93 (0.76, 1.12)	1.04 (0.77, 1.37)	0.85 (0.62, 1.16)	0.88 (0.67, 1.11)	0.98 (0.93, 1.04)

*Note:* Vitamin intake from foods and dietary supplements combined. Retinol is calculated as retinol equivalents and vitamin E as alpha‐tocopherol equivalents. Any IA, islet autoantibody positivity on two consecutive visits; IAA‐first, IA with IAA as first antibody; GADA‐first, IA with GADA as first antibody; multiple IA, repeated positivity to multiple autoantibodies, T1D, type 1 diabetes.

^a^ HR (95% CrI, credible interval) are based on joint models run separately for each nutrient. HR for 1 SD increase in age‐specific intake. Adjusted for energy intake, HLA‐DR3/4 genotype (yes, no), sex, first‐degree relative with T1D, country.

^b^ HR (95% CI, confidence interval) are based on time‐invariant cox regression model. HR for 100 μg//1000 kcal increase in intake for retinol and *β*‐carotene, and per 1 mg/1000 kcal increase for vitamin E. Adjusted for energy intake, HLA‐DR3/4 genotype (yes, no), sex, first‐degree relative with T1D, country age at seroconversion and first autoantibody.

We observed a modifying effect of country on the association between *β*‐carotene intake and risk of islet autoimmunity, suggesting an inverse association among German (HR 0.31; 95% Credible interval (CrI) 0.17, 0.61, *p* < 0.001) and direct association among Swedish (HR 2.20; 95% CrI 1.33, 3.79, *p* < 0.001) participants. We observed no vitamin intake‐HLA interactions, vitamin intake‐sex interactions, or vitamin intake‐breastfeeding interactions.

Dietary intake of retinol, *β*‐carotene, and vitamin E was not associated with progression from islet autoimmunity to T1D (Table 2). We observed no interactions for vitamin intake and age at islet autoimmunity seroconversion, first‐appearing autoantibody, country, or breastfeeding status with the risk of progression. However, we observed interaction between intakes of retinol and *β*‐carotene with sex suggesting a direct association of retinol (HR 1.070; 95% Confidence interval (CI) 1.02, 1.13, *p* = 0.008) and *β*‐carotene (HR 1.01; 95% CI 1.00, 1.02, *p* = 0.005) with progression among males but not among females: retinol (HR 0.97; 95% CI 0.97, 1.03) and *β*‐carotene (HR 1.00 95% CI 0.98, 1.01).

### 3.2 Nested Case‐Control Study

Of the 418 case children with islet autoimmunity in the original nested case‐control selection, 6 case children were excluded due to HLA ineligibility, 51 due to missing data in biomarkers, and 2 due to missing a control having biomarker data at the same visit (Supporting Information S1; Figure S1). The nested case‐control study population is described in Table 3. In the nested‐case control study, the median case age was 1.8 years for islet autoimmunity and 3.3 years for T1D.

**TABLE 3:** Characteristics of TEDDY participants in the nested case‐control study.

	Case children with islet autoimmunity *n* = 359	Matched control children *n* = 1033	Case children with type 1 diabetes *n* = 104	Matched control children *n* = 298
	*n* (%)	*n* (%)	*n* (%)	*n* (%)
Sex				
Female	160 (44.6)	459 (44.4)	47 (45.2)	131 (44.0)
Male	199 (55.4)	574 (55.6)	57 (54.8)	167 (56.0)
Clinical centre				
Colorado	52 (14.5)	151 (14.6)	15 (14.4)	42 (14.1)
Georgia	24 (6.7)	66 (6.4)	6 (5.8)	17 (5.7)
Washington	31 (8.6)	89 (8.6)	7 (6.7)	18 (6.0)
Finland	104 (29.0)	307 (29.7)	34 (32.7)	101 (33.9)
Germany	25 (7.0)	67 (6.5)	15 (14.4)	39 (13.1)
Sweden	123 (34.3)	353 (34.2)	27 (26.0)	81 (27.2)
HLA^a^				
DR3/4	193 (53.8)	371 (35.9)	59 (56.7)	115 (38.6)
Other	166 (46.2)	662 (64.1)	45 (43.3)	183 (61.4)
FDR^b^				
No	284 (79.1)	822 (79.6)	67 (64.4)	196 (65.8)
Yes	75 (20.9)	211 (20.4)	37 (35.6)	102 (34.2)

*Note:* Control children matched for family history of type 1 diabetes (FDR), clinical centre and sex.

^a^ HLA, human leucocyte antigen.

^b^ FDR, first degree relative to T1D.

#### 3.2.1 Vitamin Biomarkers

Median plasma retinol, carotenoid, tocopherol, and cholesterol levels by visit and country are presented in Supporting Information S1; Figure S5. Correlations between biomarkers are shown in Supporting Information S1; Figure S6. The carotenoid biomarkers correlated directly with each other.

**FIGURE 1:**
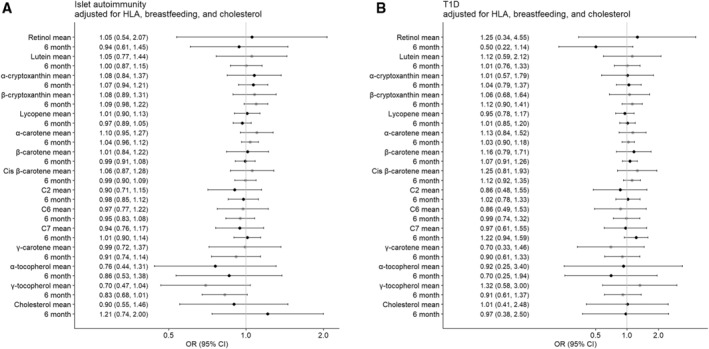
Adjusted associations between plasma retinol, carotenoids, and tocopherols and risk of islet autoimmunity (A) and type 1 diabetes (T1D) (B) in children aged 6 months to 6 years, the TEDDY nested case‐control study. Biomarkers were modelled as mean of all visits before islet autoimmunity, and at 6 months of age. Mean models include 359 matched sets for islet autoimmunity and 104 for T1D; 6‐month models include 285 for islet autoimmunity and 81 for T1D. Islet autoimmunity and persistent confirmed islet autoimmunity to at least autoantibody. Control children were matched for FDR, clinical centre, and sex. OR (95% CI, confidence interval) were based on conditional logistic regression ran separately for each biomarker. Adjusted for HLA‐DR3/4 genotype (yes, no), breastfeeding at 6 months (yes, no), and cholesterol. C2 (likely lutein metabolite), C6 (likely rubixanthin), and C7 (likely zeinoxanthin) are minor carotenoids/carotenoid metabolites.

Plasma retinol, carotenoid, and tocopherol levels were not associated with islet autoimmunity in the HLA‐adjusted (Supporting Information S1; Figure S7) nor in HLA, breastfeeding, and cholesterol‐adjusted models (Figure 1) or with further adjustments, including adjustment for long‐distance protocol. Sensitivity analyses support the use of linear models, and there was no indication of interaction with time. Further, the results are similar after the exclusion of 293 samples (85 for T1D analyses) due to failed plasma sample internal quality control. We observed interactions with country for plasma carotenoid C2 (lutein metabolite), *α*‐carotene, lutein, *α*‐cryptoxanthin, and *β*‐cryptoxanthin suggesting a tendency for a direct association of these metabolites and islet autoimmunity in the USA, indirect association in the Finnish participants, and a direct association between *β*‐cryptoxanthin and islet immunity among German participants (Supporting Information S1; Table S1). Further, we observed interaction for mean *α*‐cryptoxanthin and breastfeeding at 6 months, suggesting a non‐significant inverse association for the breastfed children (OR 0.73, 95% CI 0.49, 1.07) per one log‐unit increase in concentrations and a direct association for the non‐breastfed children (OR 1.40, 95% CI (1.01, 1.93)). We observed no sex‐biomarker interaction for risk of islet autoimmunity.

Plasma retinol, carotenoid, and tocopherol levels were not associated with T1D (Figure 1). Restricting the exposure assessment up to 1.5 years before T1D to avoid potential reverse‐causality did not affect the results.

**FIGURE 2:**
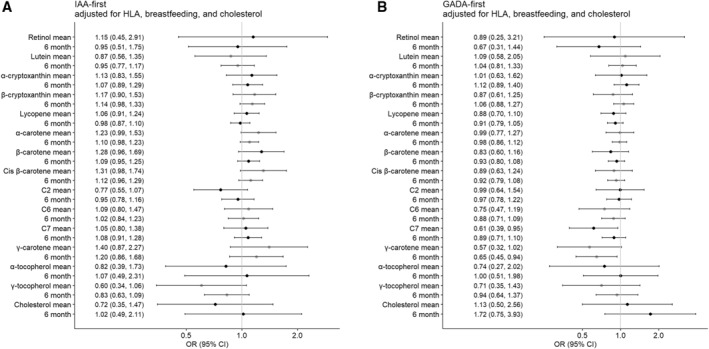
Adjusted associations between plasma retinols, carotenoids, and tocopherols and risk of IAA‐first (A) and GADA‐first (B) islet autoimmunity in children aged 6 months to 6 years, the TEDDY nested case‐control study. The number of IAA‐first cases/controls was 172/485 in mean models and 144/381 in 6‐month models. Respective numbers for GADA‐first are 120/354 and 90/249. Control children were matched for FDR, clinical centre, and sex. OR (95% CI, confidence interval) were based on conditional logistic regression ran separately for each biomarker. Adjusted for HLA‐DR3/4 genotype (yes, no), breastfeeding at 6 months (yes, no), and cholesterol. Biomarkers were modelled as means of all visits before islet autoimmunity, and at 6 months of age. C2 (likely lutein metabolite), C6 (likely rubixanthin), and C7 (likely zeinoxanthin) are minor carotenoids/carotenoid metabolites.

When studying the risk of first‐appearing autoantibody, we observed that all visits mean level of carotenoid metabolite C7 (likely zeinoxanthin) (OR 0.61, 95% CI 0.39, 0.95: *p* = 0.03) and 6‐month *γ*‐carotene (OR 0.65, 95% CI 0.45, 0.94: *p* = 0.02) were inversely associated with developing GADA‐first, adjusted for HLA, breastfeeding, and cholesterol (Figure 2). None of the biomarkers were associated with the risk of IAA‐first (Figure 2).

## 4 Discussion

In this prospective multinational birth cohort with children susceptible to T1D, dietary intake of vitamins A and E and their major biomarkers were not consistently associated with the risk of islet autoimmunity and T1D. However, we observed some GADA‐first‐specific associations, and some subgroup associations based on country, age, sex, or breastfeeding status.

This study has several strengths. First, we followed a large group of T1D susceptible children from four countries with harmonised data collection methods, including food composition database harmonisation. Second, this is the first study to explore both dietary intake and plasma biomarkers of retinol, carotenoids, and vitamin E in relation to T1D‐related outcomes. Finally, the large dataset allowed analyses of interactions and first‐appearing autoantibodies. A limitation is that due to the observational nature of the study, multiple tests, and limited power in IAA‐ and GADA‐first analyses in nested case‐control study, some of the observations may be false positive or false negative findings. Furthermore, we were not able to adjust for some factors that may affect the vitamin's bioavailability, for example, meal fat composition. As our study population includes well‐nourished, high‐T1D‐risk children from the Northern and Central Europe, and the U.S., the findings are likely to be generalisable in similar populations, but not necessarily in other populations, for example in those where vitamin deficiencies are common.

The dietary intake results indicate that vitamin E may decrease the risk of islet autoimmunity at an early age but not later. However, we observed no association between vitamin E intake and IAA‐first (the early appearing autoimmunity) or between plasma tocopherols and islet autoimmunity. The most likely mechanism of how vitamin E could affect the risk of islet autoimmunity is via its antioxidant properties, hypothesized to protect islet cells from the effects of oxidative stress, similar to ascorbic acid [5]. Vitamin E's effects may also occur locally in the gut [28], which could explain the absence of associations with circulating tocopherol biomarkers. Common food sources in infancy are infant formula and human milk, and in later childhood oils, fat spreads, nuts, seeds, cereals, dairy, meats, various fruits and vegetables, and dietary supplements.

Retinol and *β*‐carotene intake, as well as carotenoid metabolites showed both direct and inverse associations with islet autoimmunity, depending on the exposure and subgroup of participants. Of the carotenoid metabolites *γ*‐carotene and carotenoid metabolite C7 (likely zeinoxanthin), lutein, lutein metabolite, and *α*‐cryptoxanthin showed some inverse associations with islet autoimmunity. The carotenoid metabolites correlated with each other and may reflect intakes from similar food sources. We have no explanation for the observed subgroup findings, and these could be later confirmed in a second TEDDY nested case control study among older participants for which the laboratory analyses are currently ongoing. The observation that some carotenoids were associated with GADA‐first only supports the idea of heterogeneity of T1D [29]. Like tocopherols, carotenoids have antioxidant properties, which could explain some of the observed associations. The most common food sources of *β*‐carotene in children are vegetables, especially root vegetables. Lutein, on the other hand, is mostly obtained from peas, broccoli, and other green vegetables, while lycopene originates from tomato and *β*‐cryptoxanthine from citrus fruit and juices.

To conclude, retinols, carotenoids and tocopherols were not consistently associated with islet autoimmunity. However, we observed some associations in GADA‐first‐specific and subgroup‐specific analyses. This study adds to the understanding of dietary factors and their interactions with country, sex and breastfeeding in relation to T1D development.

## Author Contributions

A.G.Z, W.A.H., M.J.R., R.A.M., Å.L., J.P.K., J.M.N., I.E., and S.M.V. designed research. U.U., C.A.A., S.H., J.M.N. and I.E. conducted research. J.C. analysed data and L.H., J.L.C., U.U., I.E. and S.M.V. wrote the paper. I.E. was responsible for supervising laboratory analysis of dietary biomarkers. L.H. had primary responsibility for final content. All authors have read and approved the final manuscript.

## Funding

The TEDDY Study is funded by U01 DK63829, U01 DK63861, U01 DK63821, U01 DK63865, U01 DK63863, U01 DK63836, U01 DK63790, UC4 DK63829, UC4 DK63861, UC4 DK63821, UC4 DK63865, UC4 DK63863, UC4 DK63836, UC4 DK95300, UC4 DK100238, UC4 DK106955, UC4 DK112243, UC4 DK117483, U01 DK124166, U01 DK128847, and Contract No. HHSN267200700014C from the National Institute of Diabetes and Digestive and Kidney Diseases (NIDDK), National Institute of Allergy and Infectious Diseases (NIAID), Eunice Kennedy Shriver National Institute of Child Health and Human Development (NICHD), National Institute of Environmental Health Sciences (NIEHS), Centres for Disease Control and Prevention (CDC), and Breakthrough T1D (formerly JDRF). This work is supported in part by the NIH/NCATS Clinical and Translational Science Awards to the University of Florida (UL1 TR000064) and the University of Colorado (UL1 TR002535). The content is solely the responsibility of the authors and does not necessarily represent the official views of the National Institutes of Health. In addition, writing this paper was supported by Research Council of Finland (339922) and State funding for university‐level health research, Tampere University Hospital, Wellbeing services county of Pirkanmaa.

## Conflicts of Interest

The authors declare no conflicts of interest.

## Supporting information


Supporting Information S1

## Data Availability

The data that support the findings of this study are available in the NIDDK Central Repository (NIDDK‐CR) at https://repository.niddk.nih.gov. These data were derived from the following resources available in the public domain: The Environmental Determinants of Diabetes in the Young, https://doi.org/10.58020/y3jk‐x087.
