# Supplementary material for: Retinol, Carotenoid, and Tocopherol Intake and Status, and the Risk of Islet Autoimmunity and Type 1 Diabetes: The Environmental Determinants of Diabetes in the Young Study
Source: Diabetes Metab Res Rev. 2026 Jun 26;42(5):e70196. doi: 10.1002/dmrr.70196 (PMC13308770; doi:10.1002/dmrr.70196)
Supplement: Supplementary file 1 — Supporting Information S1 [file DMRR-42-e70196-s001.docx]

**Supplementary Materials**

**Supplementary Figure 1.** Flow chart for study participants in TEDDY cohort and nested case control study.

**Supplementary Figure 2.** Median, IQR (dark gray) absolute intakes of retinol (*μ*g), β -carotene (*μ*g), and vitamin E (mg) (left panel) and per 1000 kcal of energy intake (right panel) by visit in TEDDY cohort.

**Supplementary Figure 3** Median intake retinol, β -carotene, and vitamin E per 1000 kcal of energy intake, and by visit and breastfeeding status (left panel) and by country (right panel) in TEDDY cohort of 7,718 children and 58,529 food records.

**Supplementary Figure 4**. Vitamin E intake and risk of islet autoimmunity. Green lines present time-varying hazard ratio and its 95% CrI. The dotted horizontal line is the hazard ratio from model without interaction with time. Adjusted for energy, sex, FDR (yes, no), HLA (DR3/4, other), and country.

**Supplementary Figure 5.** Median plasma retinol, carotene, tocopherol, and cholesterol levels by visit and country in 1354 TEDDY NCC1 participants. C2 (likely lutein metabolite), C6 (likely rubixanthin), and C7 (likely zeinoxanthin) are minor carotenoids/carotenoid metabolites.

**Supplementary Figure 6.** Pearson correlations between plasma cholesterol, retinol, carotenoids, and tocopherol biomarkers in 1354 TEDDY NCC1 participants. C2 (likely lutein metabolite/zeaxanthin), C6 (likely rubixanthin), and C7 (likely zeinoxanthin) are carotenoid metabolites.

**Supplementary Figure 7.** HLA-adjusted associations between plasma retinol, carotenoids, and tocopherols and risk of islet autoimmunity (A) and type 1 diabetes (B) in children aged 6 months to 6 years, the TEDDY nested case-control study.

**Supplementary Table 1.** Country-stratified associations of mean plasma carotenoid metabolite-2, α-carotene, lutein, and α and β cryptoxanthin levels and risk of islet autoimmunity, TEDDY Study.

**The TEDDY Study Group**


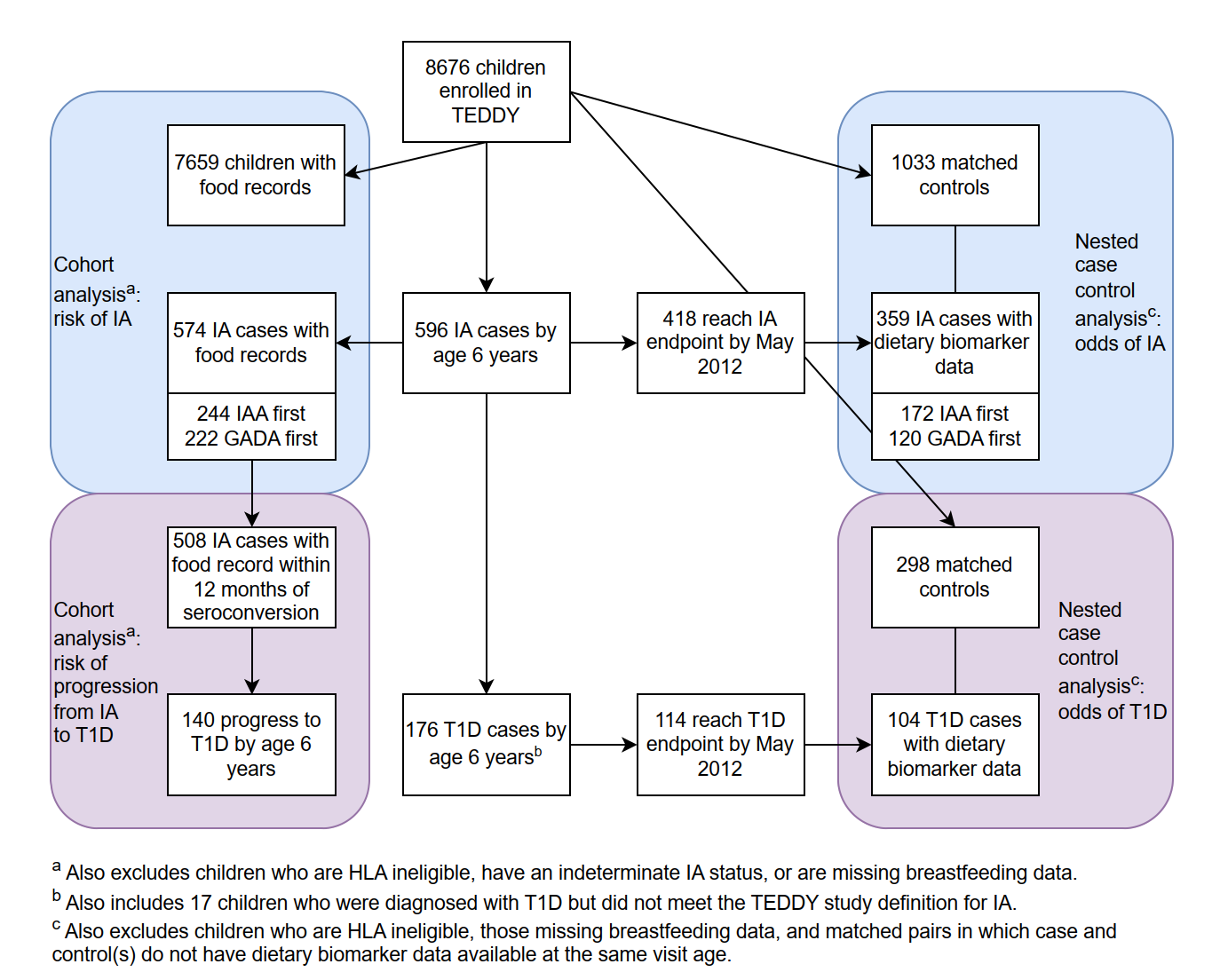
 **Supplementary Figure 1.** Flow chart for study participants in TEDDY cohort and nested case control study.

IA, islet autoimmunity; T1D, type 1 diabetes


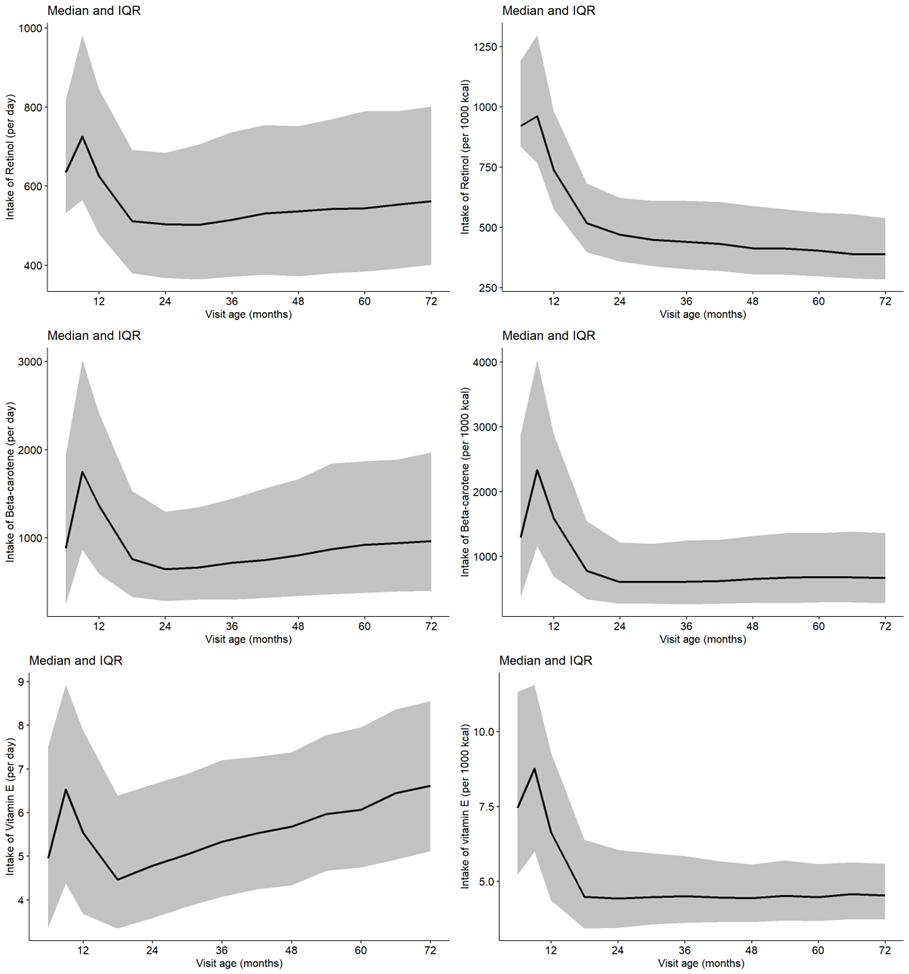


**Supplementary Figure 2.** Median, IQR (dark gray) absolute intakes of retinol (*μ*g), β -carotene (*μ*g), and vitamin E (mg) (left panel) and per 1000 kcal of energy intake (right panel) by visit in TEDDY cohort.


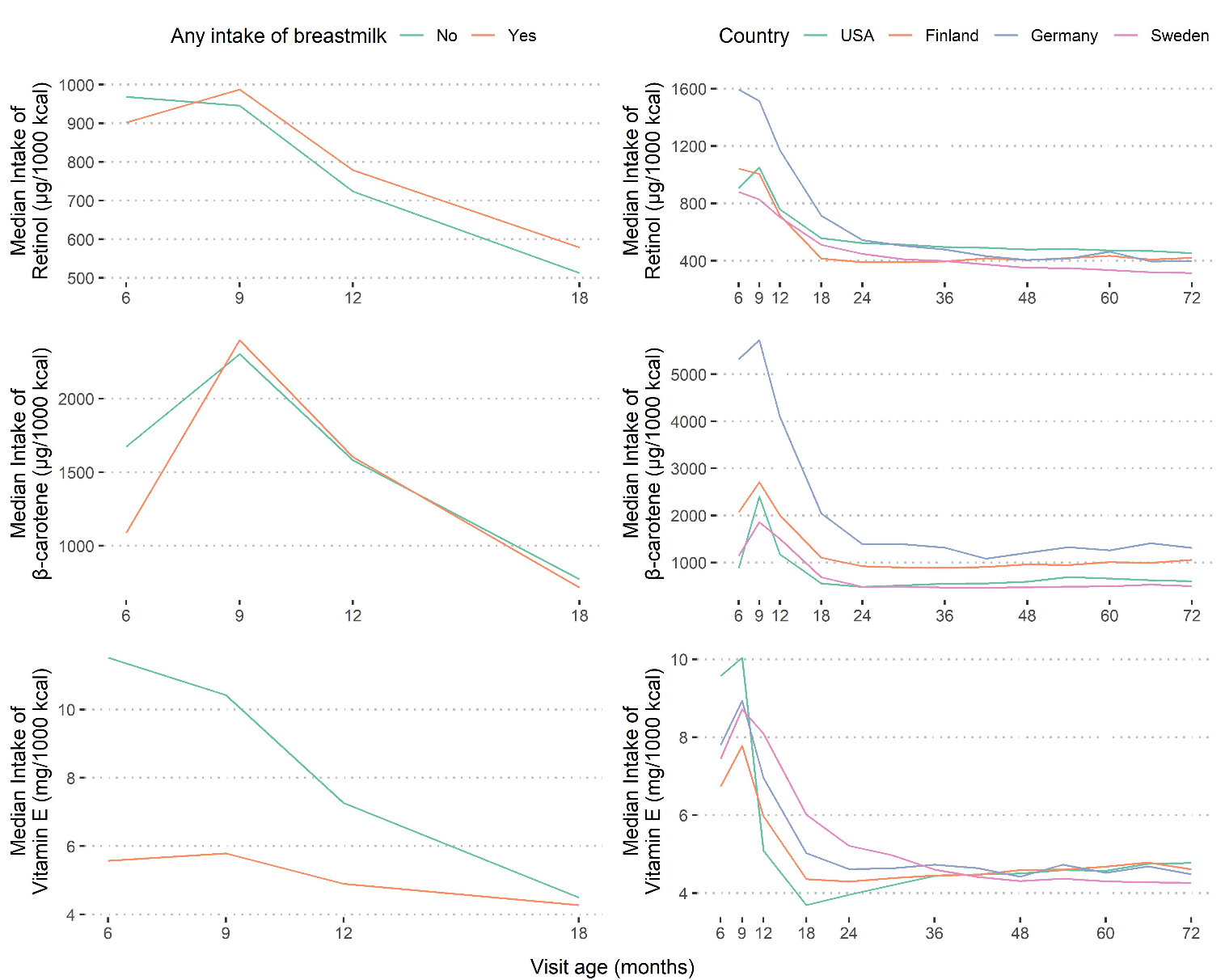


**Supplementary Figure 3** Median intake retinol, β -carotene, and vitamin E per 1000 kcal of energy intake, and by visit and breastfeeding status (left panel) and by country (right panel) in TEDDY cohort of 7,718 children and 58,529 food records.


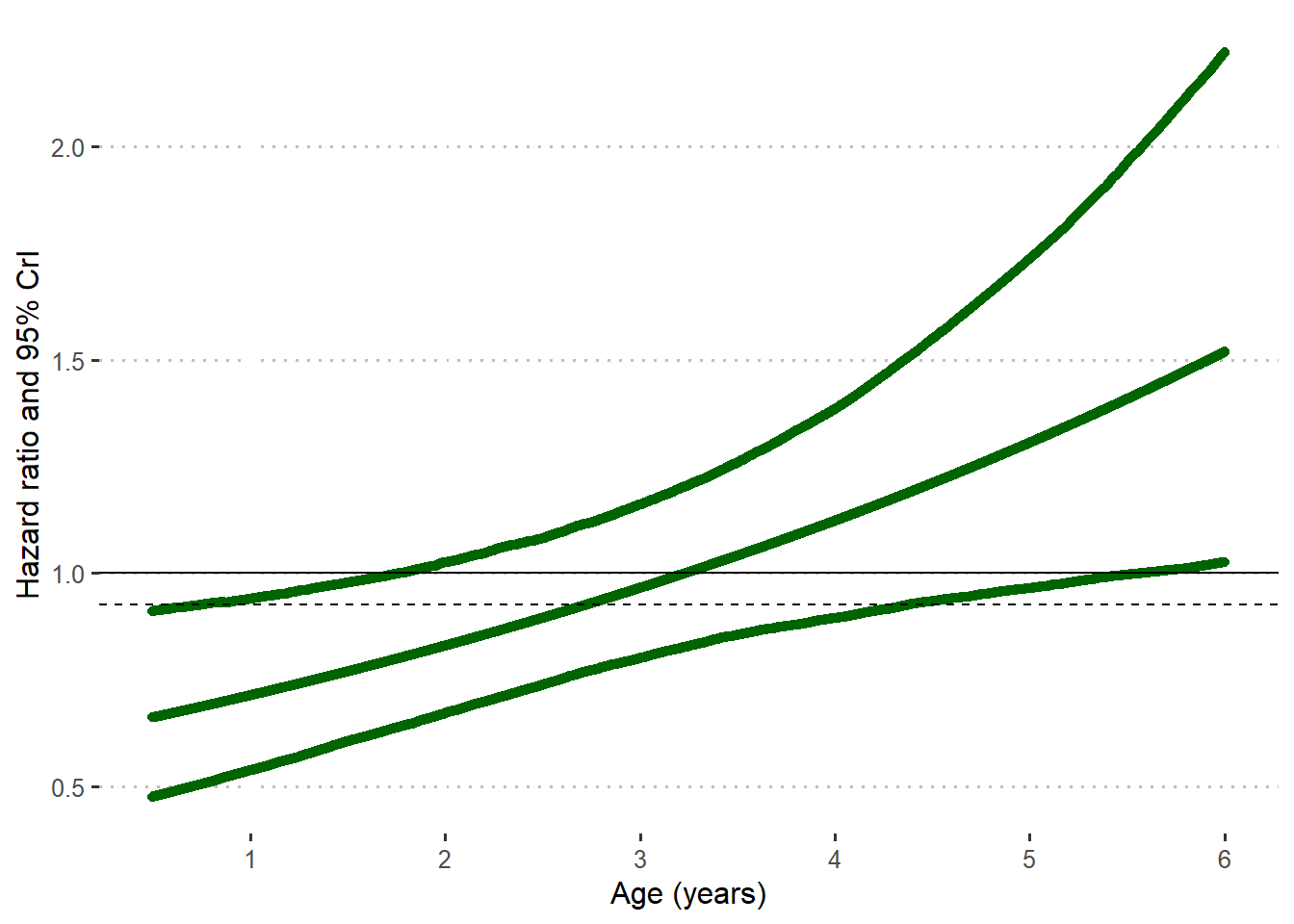


**Supplementary Figure 4**. Vitamin E intake and risk of islet autoimmunity. Green lines present time-varying hazard ratio and its 95% CrI. The dotted horizontal line is the hazard ratio from model without interaction with time. Adjusted for energy, sex, FDR (yes, no), HLA (DR3/4, other), and country.


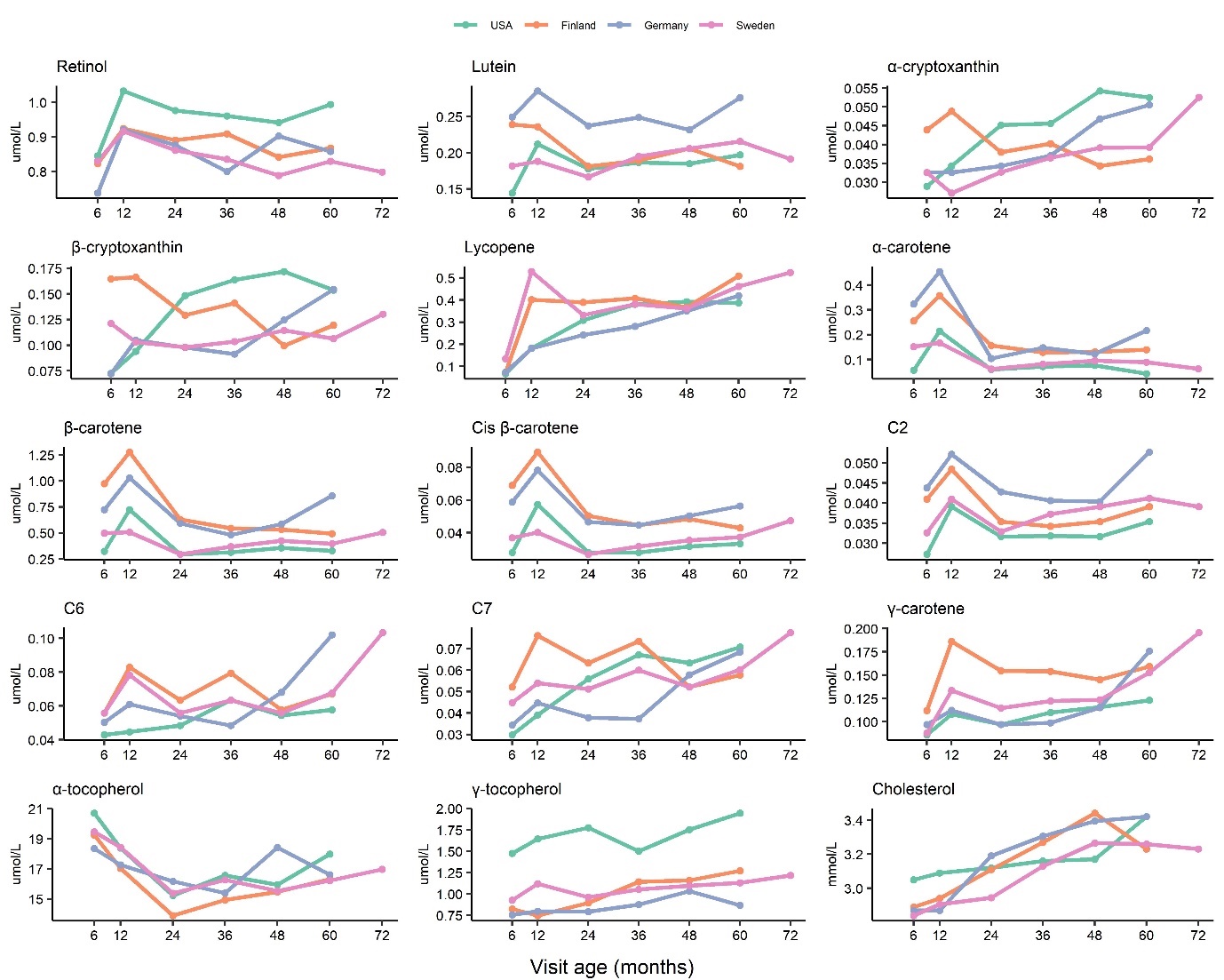


**Supplementary Figure 5.** Median plasma retinol, carotene, tocopherol, and cholesterol levels by visit and country in 1354 TEDDY NCC1 participants. C2 (likely lutein metabolite), C6 (likely rubixanthin), and C7 (likely zeinoxanthin) are minor carotenoids/carotenoid metabolites.


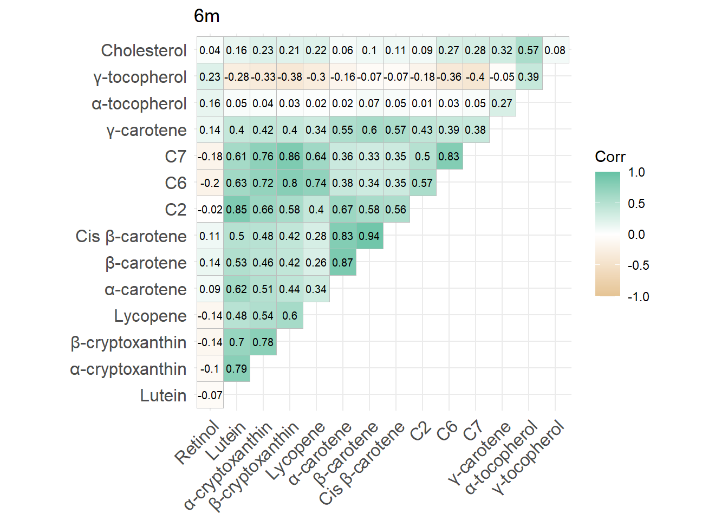

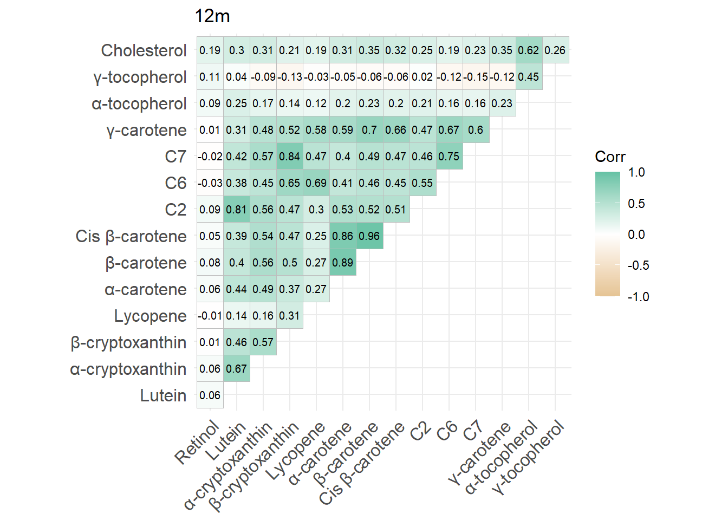


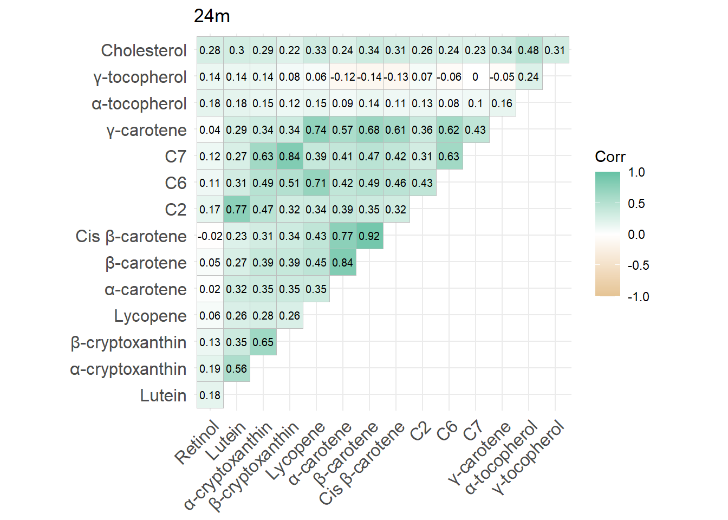

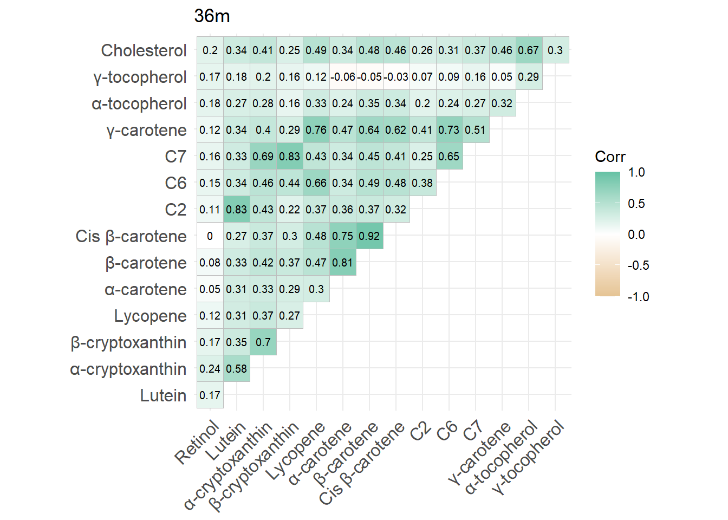


**Supplementary Figure 6.** Pearson correlations between plasma cholesterol, retinol, carotenoids, and tocopherol biomarkers in 1354 TEDDY NCC1 participants. C2 (likely lutein metabolite/zeaxanthin), C6 (likely rubixanthin), and C7 (likely zeinoxanthin) are carotenoid metabolites.

**
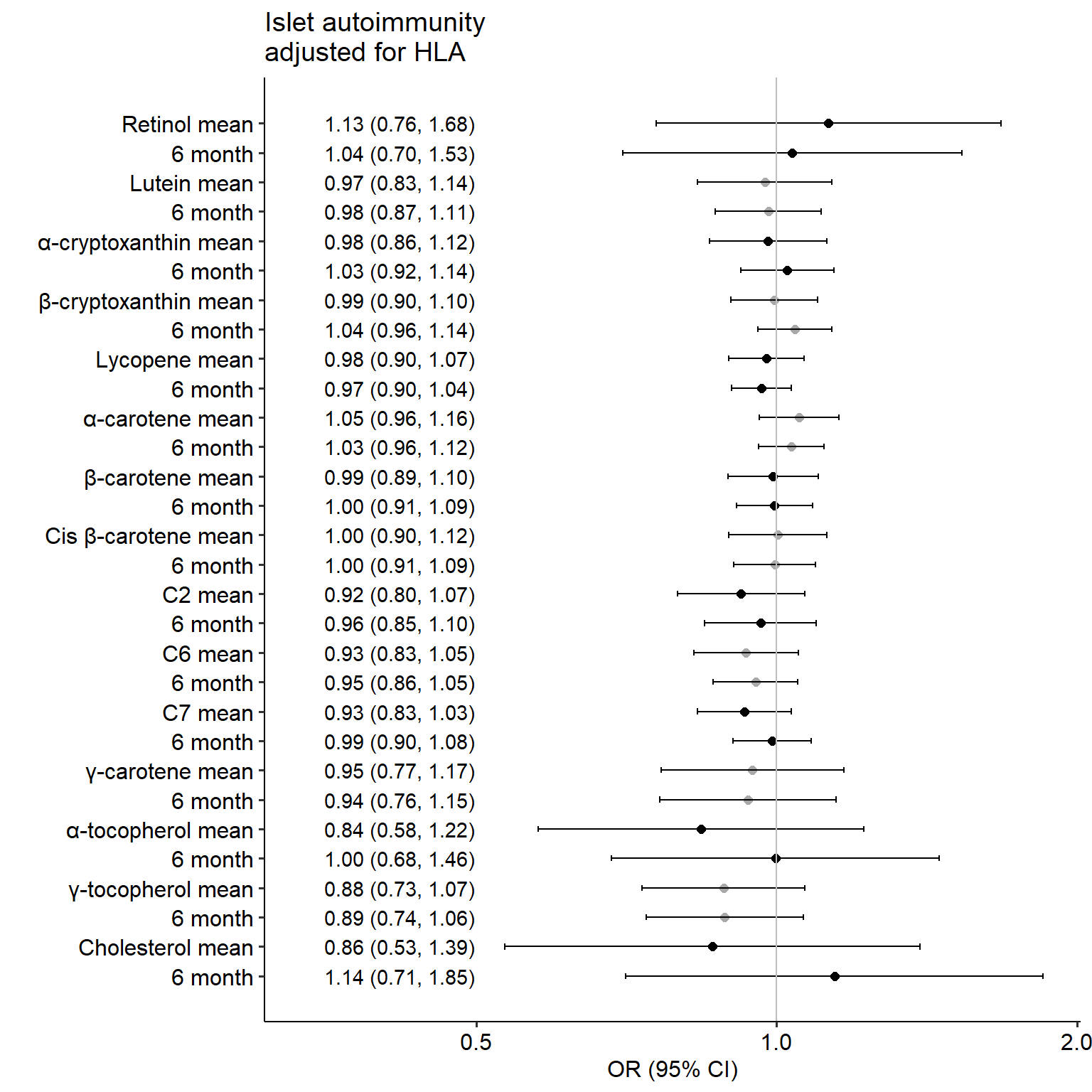

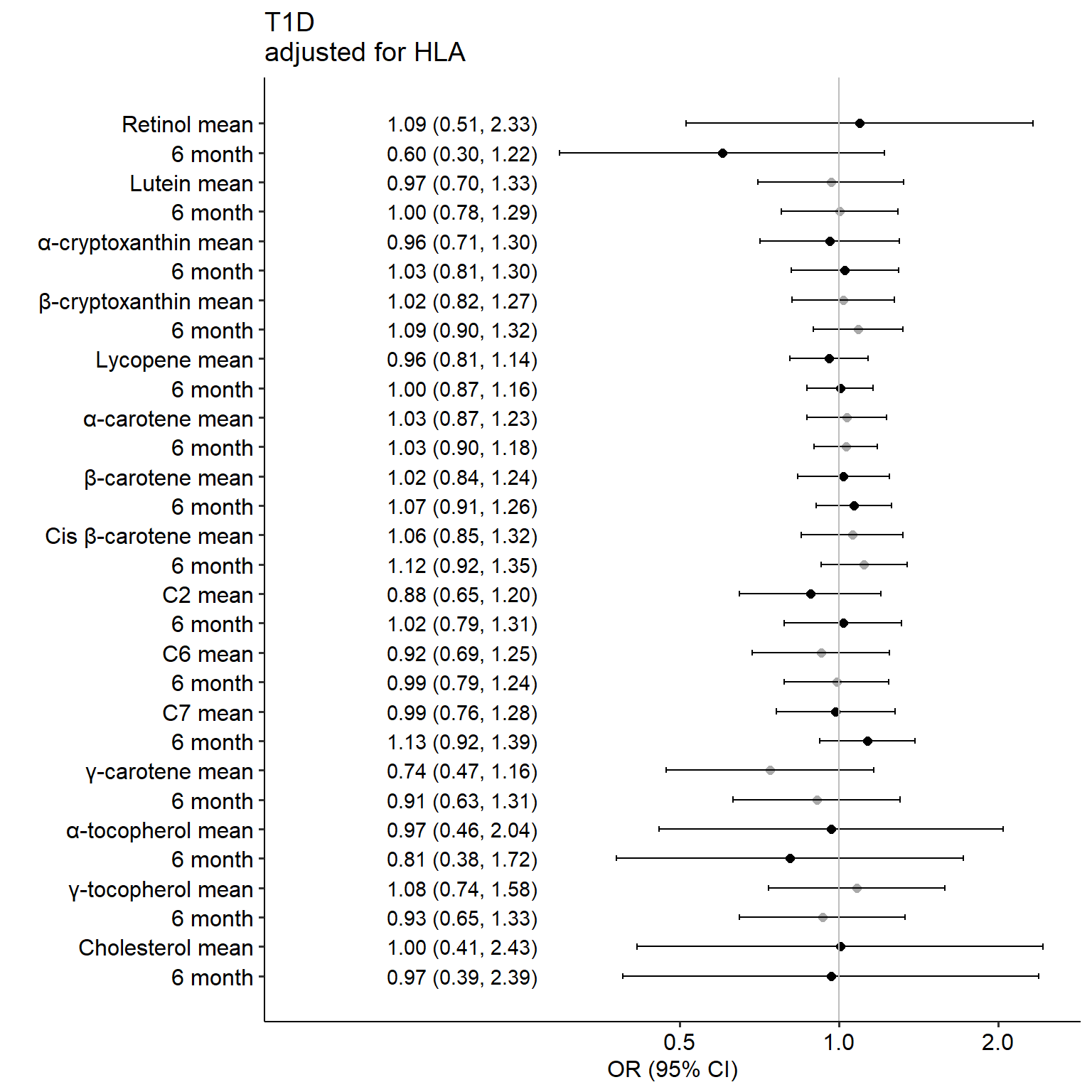
**

A

B

**Supplementary Figure 7.** HLA-adjusted associations between plasma retinol, carotenoids, and tocopherols and risk of islet autoimmunity (A) and type 1 diabetes (B) in children aged 6 months to 6 years, the TEDDY nested case-control study.

OR (95% CI, confidence interval) are based on conditional logistic regression ran separately for each biomarker. Biomarkers were modelled as means of all visits before islet autoimmunity, and as at 6 months of age. C2 (likely lutein/zeaxanthin metabolite), C6 (likely rubixanthin), and C7 (likely zeinoxanthin) are minor carotenoids present in plasma.

**Supplementary Table 1.** Country-stratified associations of mean plasma carotenoid metabolite-2, α-carotene, lutein, and α and β cryptoxanthin levels and risk of islet autoimmunity, TEDDY Study.

|  | **Carotenoid metbolite 2** | **α-carotene** | **Lutein** | **α-cryptoxanthin** | **β-cryptoxanthin** |
| --- | --- | --- | --- | --- | --- |
|  | **OR (95% CI)** | **OR (95% CI)** | **OR (95% CI)** | **OR (95% CI)** | **OR (95% CI)** |
| **US** | 1.48 (0.95, 2.32) | **1.47 (1.13, 1.90)** | 1.55 (0.91, 2.64) | 1.40 (0.92, 2.12) | 1.11 (0.82, 1.50) |
| **Finland** | **0.52 (0.31, 0.86)** | 0.86 (0.66, 1.11) | **0.53 (0.28, 0.99)** | 0.75 (0.47, 1.19) | 0.84 (0.55, 1.29) |
| **Germany** | 0.87 (0.43, 1.75) | 0.89 (0.60, 1.30) | 1.63 (0.60, 4.45) | 1.16 (0.54, 2.47) | **2.91 (1.21, 6.99)** |
| **Sweden** | 0.87 (0.58, 1.29) | 1.14 (0.84, 1.55) | 1.02 (0.58, 1.79) | 1.08 (0.72, 1.61) | 0.98 (0.73, 1.33) |

OR (95% CI, confidence interval) are based on conditional logistic regression ran separately for each biomarker.

Adjusted for HLA, breastfeeding at 6 months, and cholesterol. C2 is a carotenoid metabolite (likely lutein/zeaxanthin metabolite).

**The TEDDY Study Group**

**Colorado Clinical Center:** Marian Rewers, M.D., Ph.D., PI^1,4,6,9,10^, Kimberly Bautista^11^, Judith Baxter^8,911^, Daniel Felipe-Morales, Brigitte I. Frohnert, M.D., Ph.D.^2,13^, Marisa Stahl, M.D.^12^, Patricia Gesualdo^2,6,11,13^, Michelle Hoffman^11,12,13^, Randi Johnson, Ph.D.^2,3^, Rachel Karban^11^, Edwin Liu, M.D.^12^, Jill Norris, Ph.D.^2,3,11^, Holly O’Donnell, Ph.D.^8^, Andrea Steck, M.D.^3,13^, Kathleen Waugh^6,7,11^. University of Colorado, Anschutz Medical Campus, Barbara Davis Center for Childhood Diabetes, Aurora, CO, USA.

**Finland Clinical Center:** Jorma Toppari, M.D., Ph.D., PI^¥^1,4,10,13^, Olli G. Simell, M.D., Ph.D., Annika Adamsson, Ph.D.^^11^, Suvi Ahonen*^±§^, Mari Åkerlund*^±§^, Sirpa Anttila^µ¤^, Leena Hakola, Ph.D.*^±^, Sanni Heikura^µ¤^, Tiia Honkanen^µ¤^, Heikki Hyöty, M.D., Ph.D.*^±6^, Jorma Ilonen, M.D., Ph.D.^¥3^, Saori Itoshima, M.D.^¥^^, Sanna Jokipuu^^^, Taru Karjalainen^µ¤^, Leena Karlsson^^^, Jukka Kero, M.D., Ph.D.^¥^3, 13^, Marika Korpela^µ¤^, Jaakko J. Koskenniemi M.D., Ph.D.^¥^^, Miia Kähönen^µ¤11,13^, Mikael Knip, M.D., Ph.D.*^±^, Minna-Liisa Koivikko^µ¤^, Katja Kokkonen*^±^, Merja Koskinen*^±^, Mirva Koreasalo*^±§2^, Kalle Kurppa, M.D., Ph.D.*^±12^, Salla Kuusela, M.D. ^µ¤^, Jutta Laiho, Ph.D.*^6^, Tiina Latva-aho^µ¤^, Laura Leppänen^^^, Katri Lindfors, Ph.D.*^12^, Maria Lönnrot, M.D., Ph.D.*^±6^, Elina Mäntymäki^^^, Markus Mattila, Ph.D.*^±2^, Maija E. Miettinen, Ph.D.^§2^, Teija Mykkänen^µ¤^, Tiina Niininen^±^*^11^, Sari Niinistö, Ph.D.^§2^, Noora Nurminen^*±^, Sami Oikarinen, Ph.D.*^±6^, Hanna-Leena Oinas*^±^, Paula Ollikainen^µ¤^, Zhian Othmani^¥^, Sirpa Pohjola ^µ¤^, Jenna Rautanen^§^, Mia Rein^µ¤^, Minna Romo^^^, Juulia Rönkä^µ¤^, Nelli Rönkä^µ¤^, Noora Ruotsalainen^µ¤^, Satu Simell, M.D., Ph.D.^¥12^, Päivi Tossavainen, M.D.^µ¤^, Mari Vähä-Mäkilä^¥^, Eeva Varjonen^^11^, Riitta Veijola, M.D., Ph.D.^µ¤13^, Irene Viinikangas^µ¤^, Suvi M. Virtanen, M.D., Ph.D.*^±§2^. ^¥^University of Turku, Turku, Finland, *Tampere University, Tampere, Finland, ^µ^University of Oulu, Oulu, Finland, ^^^Turku University Hospital, Wellbeing Services County of Southwest Finland, Turku, Finland, ^±^Tampere University Hospital, Wellbeing Services County of Pirkanmaa, Tampere, Finland, ^¤^Oulu University Hospital, Wellbeing Services County of North Ostrobothia, Oulu, Finland, ^§^Finnish Institute for Health and Welfare, Helsinki, Finland.

**Georgia/Florida Clinical Center:** Richard McIndoe, Ph.D., PI^^4,10^, Desmond Schatz*, M.D.*^4,7,8^, Diane Hopkins^^11^, Michael Haller, M.D.*^13^, Melissa Gardiner^^11^, Ashok Sharma^^^, Ph.D.^^^, Laura Jacobsen, M.D.*^13^, Percy Gordon^^^, Jennifer Hosford*. ^^^Center for Biotechnology and Genomic Medicine, Augusta University, Augusta, GA, USA. *University of Florida, Pediatric Endocrinology, Gainesville, FL, USA.

**Germany Clinical Center:** Anette G. Ziegler, M.D., PI^1,3,4,10^, Ezio Bonifacio Ph.D.*, Cigdem Sanverdi, Anja Heublein, Sandra Hummel, Ph.D.^2^, Annette Knopff^7^, Melanie Köger, Sibylle Koletzko, M.D.^¶12^, Claudia Ramminger^11^, Roswith Roth, Ph.D.^8^, Jennifer Schmidt, Marlon Scholz, Joanna Stock^8,11,13^, Katharina Warncke, M.D.^13^, Lorena Müller, Christiane Winkler, Ph.D.^2,11^. Forschergruppe Diabetes e.V. and Institute of Diabetes Research, Helmholtz Zentrum München, Forschergruppe Diabetes, and Klinikum rechts der Isar, Technische Universität München, Neuherberg, Germany. *Center for Regenerative Therapies, TU Dresden, Dresden, Germany, ^¶^Dr. von Hauner Children’s Hospital, Department of Gastroenterology, Ludwig Maximillians University Munich, Munich, Germany.

**Sweden Clinical Center:** Åke Lernmark, Ph.D., PI^1,3,4,5,6,8,9,10^, Daniel Agardh, M.D., Ph.D.^6,12^, Carin Andrén Aronsson, Ph.D.^2,11,12^, Rasmus Bennet, Corrado Cilio, Ph.D., M.D.^6^, Susanne Dahlberg, Malin Goldman Tsubarah, Emelie Ericson-Hallström, Lina Fransson, Emina Halilovic, Susanne Hyberg, Berglind Jonsdottir, M.D., Ph.D.^11^, Naghmeh Karimi, Helena Elding Larsson, M.D., Ph.D.^6,13^, Markus Lundgren, M.D., Ph.D.^13^, Jessica Melin, Ph.D.^11^, Kobra Rahmati, Anita Ramelius, Falastin Salami, Ph.D., Anette Sjöberg, Evelyn Tekum Amboh, Carina Törn, Ph.D.^3^, Terese Wiktorsson. Lund University, Lund, Sweden.

*Past staff: Eva Andersson, Marie Andersson Turpeinen, Rawya Antar, Maria Ask, Jenny Bremer, Sylvia Bianconi Svensson, Ulla-Marie Carlsson, Magdalena Delikat Kulinski, Annika Fors, Ulla Fält, Thomas Gard, Joanna Gerardsson, Monika Hansen, Anna Hansson, Carina Hansson, Gertie Hansson, Elin M. Hård af Segerstad, Ph.D.^2^, Hanna Jisser, Fredrik Johansen, Linda Jonsson, Silvija Jovic, Sigrid Lenrick Forss, Barbro Lernmark, Ph.D.^8^,* Marielle Lindström, *Maria Markan, Theodosia Massadakis, Marlena Maziarz, Ph.D., Zeliha Mestan, Maria Månsson Martinez, Caroline Nilsson, Emma Nilsson, Yohanna Nordh, Karin Ottosson, Sara Rang, Anna Rosenquist, Monika Sedig Järvirova, Sara Sibthorpe, Birgitta Sjöberg, Ulrika Swartling Ph.D.^8^, Erika Trulsson,* Ulrika Ulvenhag, *Anne Wallin, Ingrid Wigheden,* Åsa Wimar, *Sofie Åberg.*

**Washington Clinical Center:** William A. Hagopian, M.D., Ph.D., PI^^1,3,4,6,7,10,12,13^, Michael Killian*^6,7,11,12^, Claire Cowen Crouch*^11,13^, Jennifer Skidmore*^2^, Ben Kim*, Cody McCall*, Arlene Meyer*, Jared Radtke*, Shreya Roy*. ^^^Indiana University, Indianapolis, IN, USA. *Pacific Northwest Research Institute, Seattle, WA, USA.

**Pennsylvania Satellite Center:** Dorothy Becker, M.D., Margaret Franciscus, MaryEllen Dalmagro-Elias Smith^2^, Ashi Daftary, M.D., Mary Beth Klein, Chrystal Yates. Children’s Hospital of Pittsburgh of UPMC, Pittsburgh, PA, USA.

**Data Coordinating Center:** Jeffrey P. Krischer, Ph.D., PI^1,4,5,9,10^, Rajesh Adusumali, Sarah Austin-Gonzalez, Maryouri Avendano, Sandra Baethke, Brant Burkhardt, Ph.D.^6^, Martha Butterworth^2^, Nicholas Cadigan, Joanna Clasen, Ph.D., Kevin Counts, Laura Gandolfo, Jennifer Garmeson, Veena Gowda, Shu Liu, Xiang Liu, Ph.D.^2,3,8,13^, Kristian Lynch, Ph.D. ^6,8^, Jamie Malloy, Lazarus Mramba, Ph.D.^2^, Cristina McCarthy^11^, Hemang M. Parikh, Ph.D.^3,8^, Cassandra Remedios, Chris Shaffer, Susan Smith^11^, Noah Sulman, Ph.D., Roy Tamura, Ph.D.^1,2,11,12,13^, Dena Tewey, Henri Thuma, Michael Toth, Ulla Uusitalo, Ph.D.^2^, Kendra Vehik, Ph.D.^4,5,6,8,13^, Ponni Vijayakandipan, Melissa Wroble, Jimin Yang, Ph.D., R.D.^2^, Kenneth Young, Ph.D. *Past staff: Michael Abbondondolo, Lori Ballard, Rasheedah Brown, David Cuthbertson, Stephen Dankyi, Christopher Eberhard, Steven Fiske, David Hadley, Ph.D., Kathleen Heyman, Belinda Hsiao, Christina Karges, Francisco Perez Laras, Hye-Seung Lee, Ph.D., Qian Li, Ph.D., Colleen Maguire, Wendy McLeod, Aubrie Merrell, Steven Meulemans, Jose Moreno, Ryan Quigley, Laura Smith, Ph.D.* University of South Florida, Tampa, FL, USA.

**Autoantibody Reference Laboratories:** Liping Yu, M.D.^^5^, Dongmei Miao, M.D.^^^, Kathleen Gillespie*^5^, Kyla Chandler*, Olivia Pearce*, Sarah Stollery*, Elinor Balch*, Hanah Batholomew*, Zahra Hashmi*. ^^^Barbara Davis Center for Childhood Diabetes, University of Colorado Denver, *Bristol Medical School, University of Bristol, UK.

**Dietary Biomarkers Laboratory:** Iris Erlund, Ph.D.^2^, Terhi Vihervaara Ph.D., Inga Liukko, Linda Patrikainen, Outi Ruotsalainen. Institute for Nutrition and Health Research, Helsinki, Finland.

**Genetics Laboratory:** Stephen S. Rich, Ph.D.^3^, Wei-Min Chen, Ph.D.^3^, Suna Onengut-Gumuscu, Ph.D.^3^, Emily Farber, Rebecca Roche Pickin, Ph.D., Jonathan Davis, Jordan Davis, Dan Gallo, Jessica Bonnie, Paul Campolieto. Center for Public Health Genomics, University of Virginia, Charlottesville, VA, USA.

**HLA Reference Laboratory:** William Hagopian^3^, M.D., Ph.D., Jared Radtke. Pacific Northwest Research Institute, Seattle, WA, USA. (Previously Henry Erlich, Ph.D.^3^, Steven J. Mack, Ph.D., Anna Lisa Fear. Center for Genetics, Children’s Hospital Oakland Research Institute.)

**Repository:** Chris Deigan. NIDDK Biosample Repository at Fisher BioServices, Rockville, MD, USA. (Previously Ricky Schrock, Polina Malone, Sandra Ke, Niveen Mulholland, Ph.D.)

**Project scientist:** Beena Akolkar, Ph.D.^1,3,4,5,6,7,9,10^. National Institutes of Diabetes and Digestive and Kidney Diseases, Bethesda, MD, USA.

**Other contributors:** Thomas Briese, Ph.D.^6^, Columbia University, New York, NY, USA. Todd Brusko, Ph.D.^5^, University of Florida, Gainesville, FL, USA. Teresa Buckner, Ph.D.^2^, University of Northern Colorado, Greeley, CO, USA. Suzanne Bennett Johnson, Ph.D.^8,11^, Florida State University, Tallahassee, FL, USA. Eoin McKinney, Ph.D.^5^, University of Cambridge, Cambridge, UK. Tomi Pastinen, M.D., Ph.D.^5,6^, The Children’s Mercy Hospital, Kansas City, MO, USA. Steffen Ullitz Thorsen, M.D., Ph.D.^2^, Department of Clinical Immunology, University of Copenhagen, Copenhagen, Denmark, and Department of Pediatrics and Adolescents, Copenhagen University Hospital, Herlev, Denmark. Eric Triplett, Ph.D.^6^, University of Florida, Gainesville, FL, USA.

***Committees:***

^1^Ancillary Studies, ^2^Diet, ^3^Genetics, ^4^Human Subjects/Publicity/Publications, ^5^Immune Markers, ^6^Infectious Agents, ^7^Laboratory Implementation, ^8^Psychosocial, ^9^Quality Assurance, ^10^Steering, ^11^Study Coordinators, ^12^Celiac Disease, ^13^Clinical Implementation.
